# Web-Based and mHealth Technologies to Support Self-Management in People Living With Type 2 Diabetes: Validation of the Diabetes Self-Management and Technology Questionnaire (DSMT-Q)

**DOI:** 10.2196/18208

**Published:** 2020-07-09

**Authors:** Laura Kelly, Crispin Jenkinson, David Morley

**Affiliations:** 1 Health Services Research Unit Nuffield Department of Population Health University of Oxford Oxford United Kingdom; 2 Harris Manchester College Oxford United Kingdom

**Keywords:** mHealth, self-care, type 2 diabetes, self-monitoring, questionnaire

## Abstract

**Background:**

A growing number of web-based and mobile health (mHealth) technologies have been developed to support type 2 diabetes self-management. Little is known about individuals’ experiences with these technologies and how they support self-management. Appropriate tools are needed to understand how web-based and mHealth interventions may impact self-management.

**Objective:**

This study aimed to develop an instrument, the Diabetes Self-Management and Technology Questionnaire (DSMT-Q), to assess self-management among people living with type 2 diabetes who use web-based and mHealth technologies.

**Methods:**

A total of 36 candidate questionnaire items, drafted previously, were refined using cognitive debriefing interviews (n=8), expert consultation, and public patient involvement feedback. Item reduction steps were performed on survey data (n=250), and tests of validity and reliability were subsequently performed.

**Results:**

Following amendments, patients and experts found 21 items relevant and acceptable for inclusion in the instrument. Survey participants included 104 (41.6%) women and 146 (58.4%) men. Two subscales with high construct validity, internal consistency, and test-retest reliability were identified: “Understanding individual health and making informed decisions” and “Confidence to reach and sustain goals.”

**Conclusions:**

Analyses confirmed good psychometric properties in the DSMT-Q scales. This tool will facilitate the measurement of self-management in people living with type 2 diabetes who use web-based or mHealth technologies.

## Introduction

In 2018, just over 3.8 million people in the United Kingdom were diagnosed with diabetes [1], an increase of 2.4 million since 1996 [2]. A total of 90% of those diagnosed with diabetes are thought to have type 2 diabetes (diabetes mellitus), with a further 1 million estimated to be unaware they have the condition [2]. Complications that may need to be managed include gastroparesis, painful diabetic neuropathy, autonomic neuropathy, foot problems, kidney disease, erectile dysfunction, and eye disease [3]. Complications arising from this long-term condition can be avoided mainly through supporting patients to manage their condition through, for example, through achieving glycemic control, through education and/or through lifestyle changes. Nevertheless, they are estimated to cost the National Health Service over GBP £7 billion (US $8.7 billion) per year in direct costs [4].

People living with type 2 diabetes need to be supported to manage their condition, improve well-being, and prevent diabetes-related complications from arising. Key management priorities for UK health services include patient education, dietary advice, blood glucose management, and drug treatment [3]. While some areas of routine care (for example, therapy changes) may need to be implemented during face-to-face interactions, digital health care can also be adopted outside these interactions (for example, when promoting adherence and providing peer support) [5]. Some evidence indicates that web-based and mHealth technologies can be used successfully to enable patients to access information, individualize management, track progress and reach personalized goals and, facilitate communication with health professionals or peers [6]. The use of technology has led to improvements in physical activity, diet, problem-solving, and blood glucose control [7-12]; however, some evidence suggests that there may be aspects of self-care that are best-supported face-to-face [13]. In the long term, it may be that self-management technologies (for example, a mobile app targeting blood glucose control), are most effective when they are used interactively with a professional health care team [14].

Despite the availability of many mHealth self-management technologies, minimal evidence exists around their effectiveness, particularly concerning longer-term outcomes [15]. Evaluations of mobile apps aimed at encouraging behavior change predominantly focus on content evaluation and few measure effectiveness [16]. This gap is particularly evident for diabetes-related apps where content or usability is typically evaluated using self-developed checklists or through user feedback [16]. Many instruments used to measure the effectiveness of diabetes-related web-based or mHealth interventions have poor psychometric properties, do not meet guidelines promoted by regulatory bodies (for example, by not including the patient during development [17,18]) and may lack sensitivity to the effects of web-based and mHealth technologies as they were developed before their existence [8].

Assessing the effectiveness of technologies supporting self-management requires suitable instruments that (1) are appropriate for use with people living with type 2 diabetes, (2) includes user perspectives throughout development, and (3) is sensitive to the impact of web-based and mHealth technologies. A truly useful instrument would also be suitable for use in a comparator group not receiving the intervention. This study, therefore, aimed to develop a new measure, the Diabetes Self-Management and Technology Questionnaire (DSMT-Q), to assess self-management among people living with type 2 diabetes using web-based or mHealth technologies.

The content of the DSMT-Q was informed by a previous study that undertook in-depth qualitative interviews (n=15) with people living with type 2 diabetes in order to explore experiences of using web-based and mHealth technologies to manage their health [19]. The analysis identified seven themes as important to participants when using technology to support self-management. These themes were termed: information, understanding individual health and personal data, reaching and sustaining goals, minimizing disruption to daily life, reassurance, communicating with health care professionals, and coordinated care (see Kelly et al [19] for further details). Draft questionnaire items were constructed to reflect the seven themes, forming an item pool of 36 candidate items for the new questionnaire. The 36 questionnaire items were arranged in two parts. The first 22 items asked about the management of type 2 diabetes and the use of web-based or mHealth technology, while a further 14 items asked about the extent to which a specific technology helped to manage aspects of diabetes. This paper reports the item refinement and psychometric validation of the candidate items.

## Methods

### Design and Ethics

A mixed methods study, Phase 1 aimed to refine the 36 candidate DSMT-Q items drafted previously using cognitive debrief interviews, expert consultation, and public patient involvement (PPI) feedback. Phase 2 carried out a psychometric validation of the remaining candidate items using appropriate quantitative methods. Ethical approval for this research was granted by the Medical Sciences Inter Divisional Research Ethics Committee of the University of Oxford (reference R59651/RE001).

### Phase 1: Patient, Expert, and Public Item Refinement

#### Cognitive Interviews of People Living With Type 2 Diabetes

Thirty-six candidate items were pretested for ease of completion and understanding among people living with diabetes to ensure that items superficially made sense [20], and provided further support for content validity through ensuring that each theme identified in the qualitative interviews [19] was represented through the item content [21]. Participants were probed about their understanding of the proposed items and each item’s relevance to self-managing health and the use of technology [21,22]. In cases where items were ambiguous or repetitive, they were amended or removed.

#### Study Participants and Procedure

Participants were aged 18 or over with a (self-reported) clinical diagnosis of type 2 diabetes and experience of using one or more diabetes-related web-based or mHealth technology. Participants who had previously taken part in an in-depth interview [19] to inform items and who consented to be contacted were emailed a participant information sheet. On agreeing to take part, participants could ask any questions about the research, asked to complete an online consent form, and given a link to the draft online survey containing the candidate items. Participants were given a GBP £20 (US $24.92) voucher for their participation.

#### Interviews

Interviews were recorded and carried out over the telephone using a verbal probing method of cognitive interviewing to allow respondents an opportunity to give uninterrupted answers, which was then followed by a focused interview [23]. During the focused interview, participants were reminded of their answers to each item, and to gain a deeper understanding of their responses, the reasoning behind their answers was explored [24].

#### Analysis

Participant comments were summarized and collated in an Excel document (Microsoft) according to each instruction and questionnaire item, allowing within-case (how the item fits within the questionnaire as a whole) and between-case (interpretation and consistency of items across the sample) analysis. Interpretation difficulties or inconsistencies were discussed among authors, amended where appropriate, and retested.

#### Expert Consultation

An expert panel consisting of three survey development and patient-reported outcome experts, one survey expert and user engagement manager for a national diabetes program, two diabetes experts specializing in digital health, one professor of diabetic medicine, and one consultant physician in diabetes were invited to review the candidate DSMT-Q items via email. Consulting experts sought to evaluate items from both health professionals and survey developers’ perspectives. Comments and feedback were received via email, and items amended where appropriate after discussion among the authors.

#### PPI

PPI representatives who were members of a volunteer list held by the Nuffield Department of Primary Care Health Sciences Coordinator of Patient & Public Involvement were emailed an invite to take part in questionnaire feedback. Representatives were required to have type 2 diabetes, but as questions were designed to be answerable to both those who use technology to manage their health and those who do not, they were not required to have experience using technology to manage their health. Feedback was given over the telephone after representatives had been allowed to review the online survey.

### Phase 2: Psychometric Validation

Two web-based surveys were formatted using Qualtric’s survey software. Survey 1 included the refined DSMT-Q (21 items), the Diabetes Self-Efficacy Scale (DSES) [25], two questions on the use of technology and additional demographic questions.

The first question in Survey 1 asked the respondent if they had experience using web-based or mHealth technologies to manage their diabetes. They were shown an appropriate preamble to the DSMT-Q items: ‘Think about the management of your type 2 diabetes over the *past four weeks*’ or ‘Think about the management of your type 2 diabetes, including your use of web-based or mobile technology, over the *past four weeks*.’ All item stems remained the same regardless of the questionnaire preamble, and all responses were collated for item analysis.

The DSES is an eight-item scale to assess self-efficacy among people living with diabetes. The Diabetes Self-Efficacy Scale was developed for a randomized trial assessing community-based peer-led diabetes self-management [25]. Items were based on earlier chronic-disease self-efficacy scales [26]. The internal consistency of items is high (α=.85), and the scale demonstrates good test-retest reliability (intraclass correlation coefficient [ICC]=0.80) [25].

Survey 2 aimed to assess the test-retest reliability of the DSMT-Q. The DSMT-Q, together with a transition item (whether the respondent’s health has changed in the last two weeks), was therefore administered 2 weeks after Survey 1 had been completed.

### Procedure

#### Study Participants and Recruitment

Participants were aged 18 or over with a (self-reported) clinical diagnosis of type 2 diabetes. Participants were recruited through a professional survey recruitment company. Eligible participants were provided with a participant information sheet and asked to confirm their consent to take part. Participants who confirmed they might be contacted again regarding the study while completing Survey 1 were sent an email 2 weeks later asking them to complete Survey 2.

We aimed to recruit 250 participants to complete Survey 1. Estimates suggest that meaningful psychometric tests require at least three times as many respondents as items [27], making this a conservative (large) sample size.

#### Analysis

Descriptive statistics were used to present demographic data. DSMT-Q items were subjected to several initial data checks to confirm their suitability for inclusion in further analysis. Decision rules for item removal included items with high floor and ceiling effects (>40% of respondents selecting one of the extreme response options) [28,29] and items demonstrating a large number of weak correlations (<0.2) with other items. Exploratory factor analysis (EFA) was performed to group items into conceptually sound sub-scales. Suitability of using EFA on the dataset was assessed through performing the Bartlett Test of Sphericity (*P*<.05) [30] and calculating the Kaiser–Meyer–Olkin (KMO) statistic which has a recommended value of above 0.6 [31]. Factors with Eigenvalues >1 were rotated using an oblique, Direct Oblimin, rotation so that axes were not restricted to right angles, hence allowing correlation between the factors [32,33]. While both the Structure and Pattern matrices were used in interpreting output, and the Structure matrix offered primary guidance for interpretation [34].

Once domain structures were finalized, sub-scales floor and ceiling effects (>20% of responses scoring 0 or 100) were examined, and population characteristics were explored to identify potential covariate factors impacting the final scales. Convergent validity was examined using Pearson correlation coefficients (r) to compare relationships between the DSMT-Q sub-scales and the DSES [33,35]. The DSES was hypothesized to have moderate correlations with DMST-Q scores. Internal consistency, an indication of a scale’s reliability, was evaluated using the Cronbach alpha statistic (>0.7) [36]. External reliability was assessed using the test-retest procedure with the use of the ICC statistic to assess the stability of the scores [37].

## Results

### Phase 1

#### Patient Interviews

Eight participants took part in two rounds of cognitive interviews. In the first round, participants (n=4) considered most questionnaire content to be relevant to the management of type 2 diabetes; however, considerable changes needed to be made to the arrangement of the items. Participants found it challenging to respond to the second set of mHealth specific items as they did not typically use one specific technology (for example, one mobile app) in isolation. Furthermore, many questionnaire items did not apply to every technology used, and it was, therefore, difficult for them to determine how they should respond. The second part of the questionnaire was removed, resulting in 12 items being deleted and 2 items, covering topics not already present in the first part, being restructured and included. Two further items, which asked about health care services, were deleted from part one due to participant feedback stating they were unrelated to the personal management of their type 2 diabetes. Twenty-two items were therefore retained.

Five items were amended following participant feedback. Two were amended to improve comprehension and clarity, one was amended to make it more suitable to the response options, one was changed to be more specific and capture the intended meaning better, and one item was changed to prevent duplication with a previous item.

In the second round of cognitive interviews, following participant (n=4) feedback, four items were deleted as they were considered to duplicate existing content. Five items were amended to improve clarity, and one item was revised to apply to a broader population. Two items were considered too broad and were made into four items to improve accuracy. This process resulted in 20 items for expert consultation.

#### Expert Consultation

Twenty items were circulated to the expert group. Following feedback, two items were amended to improve language for low literacy groups. One item was amended to be more inclusive to a broader range of people. Six items were amended to improve clarity, and one item was split into two items to try and find the best way to capture reassurance, resulting in 21 items.

#### Public Patient Involvement (PPI)

PPI representatives (n=4) gave feedback on the 21-item questionnaire. All items were understood by representatives; however, some showed a preference for the further granularity of items. For example, one representative expressed a wish to have separate questions for how easily they can monitor their blood glucose levels, diet, and exercise. This change was omitted due to the likelihood of high frequencies of not applicable or missing data.

### Phase 2

#### Characteristics

Survey 1 participants included 104 (41.6%) women and 146 (58.4%) men. The average age was 55.9 years old (SD 16.4, range 69 years). The modal time since diagnosis of type 2 diabetes was between one and five years ago (n=90, 36%). Most participants (n=232, 92.8%) described themselves as White British. Further sample characteristics can be viewed in Table 1.

**Table 1:** Participant characteristics.

Characteristic		Value
**Sex, n (%)**		
	Male: Female	146 (58.4): 104 (41.6)
**Age (years)**		
	Mean (SD, range)	55.9 (16.4, 19-88 years)
**Time since diagnosis, n (%)**		
	<12 months	27 (10.8)
	1-5 years	90 (36.0)
	6-10 years	56 (22.4)
	>10 years	77 (30.8)
**Ethnic group, n (%)**		
	White British	232 (92.8)
	White (other)	5 (2.0)
	Black African	4 (1.6)
	Asian	4 (1.6)
	Mixed race	3 (1.2)
	Other	2 (0.8)

#### Item Reduction and Scale Development

Six items were removed due to ceiling effects of greater than 40%. The KMO value for the remaining 15 items exceeded the recommended value of 0.6 (KMO= 0.90), and the Bartlett Test of Sphericity reached statistical significance (*P*<.01), indicating there was a correlation between the items. Three factors were initially extracted, explaining 69.1% of the variance. One factor consisted of two items and had a poor Cronbach alpha level of 0.40. These two items were removed, and a second-factor rotation extracted two factors, explaining 63.6% of the variance. See Table 2 for the factor structure and loadings. Factor 1, entitled *Understanding individual health and making informed decisions,* consisted of seven items and had a Cronbach alpha level of 0.90. The second factor, entitled *Confidence to reach and sustain goals*, consisted of six items and had a Cronbach alpha level of 0.88.

**Table 2:** DSMT-Q factors and item loadings on the Structure matrix.

Item	Factor loading^a^	
	1	2
I can easily monitor important information about my diabetes (for example, my blood glucose levels, diet, or exercise).	0.813	0.645
I am able to make sense of any information that I monitor (for example, my blood glucose levels, diet, or exercise).	0.812	0.639
I feel informed when making decisions about the management of my diabetes.	0.804	0.553
I am aware of the potential outcomes of any actions I take when managing my diabetes (for example, when taking medications or choosing foods to eat).	0.804	0.479
I have access to relevant information about my diabetes.	0.796	0.443
I can usually identify the reasons behind any changes to my blood glucose levels.	0.757	0.441
I understand how my body reacts to exercise.	0.728	0.612
I feel reassured that I am managing my diabetes well.	0.546	0.865
I think my diabetes is under control.	0.509	0.856
I can achieve any personal goals I set when managing my diabetes.	0.491	0.807
I am motivated to carry out routines to manage my diabetes (for example, take medication, exercise).	0.584	0.775
I know when to take action to maintain my desired blood glucose levels.	0.705	0.726
I feel motivated to play an active role in my diabetes management.	0.564	0.627

^a^Rotation Method: Oblimin with Kaiser Normalization.

#### Scale Distributions and Validation

Each scale was transformed to a 0-100 metric, where 0 indicated low levels of self-management, and 100 indicated high levels of self-management of type 2 diabetes. Scale scores were calculated by summing the final response values in each sub-scale and dividing the summed score by the maximum scale score. The raw scale score was then transformed into a 0-100 metric by multiplying the raw score by 100. Scale distribution statistics are reported in Table 3. Neither scale exhibited floor or ceiling effects, which was considered to be >20% of responses, achieving the minimum or maximum score. Minimal respondents achieved scores of 0, while 10.8% (n=27) of Factor 1 scores and 10.4% (n=26) of Factor 2 scores achieved the maximum score of 100.

**Table 3:** Scale score descriptive statistics (N=250).

Scale	Minimum	Maximum	Mean (SD)	Skewness	Kurtosis	ICC^a^, n=113	*P* value
Factor 1: Understanding Individual Health and making informed decisions	0	100	75.3 (18.1)	–0.89	1.19	0.89	<.001
Factor 2: Confidence to reach and sustain goals	0	100	75.4 (17.7)	–0.95	1.43	0.86	<.001
DSES^b^	0	10	7.7 (1.9)	–1.01	1.00	N/A^c^	N/A

^a^Absolute agreement.

^b^Diabetes Self-Efficacy Scale.

^c^N/A: not applicable.

Relationships between each DSMT-Q scale and a range of potential covariate factors were examined. No significant differences were found for either sex (Factor 1: t_248_=–0.54, *P*=.59 and Factor 2: t_248_=–0.35, *P*=.72) or age (n=248, Factor 1: *r*=–0.11, *P*=.10 and Factor 2: *r*=–0.06, *P*=.31) among either sub-scale scores. Nonparametric tests to examine sex (Mann-Whitney *U* test of significance; Factor 1, *P*=.59, Factor 2=0.71) and age (n=248; Factor 1: ρ=–1.37, *P*=.03, Factor 2: ρ=–0.09, *P*=.15) also demonstrated no significant differences. No significant differences were observed for time since diagnosis for using parametric (analysis of variance; Factor 1: *F_246_*=2.14, *P*=.10, Factor 2: *F_246_*=1.891, *P*=.13) or nonparametric tests (Kruskal-Wallis k independent samples; Factor 1: *P*=.13, Factor 2: *P*=.15).

Relationships between the DMST-Q scales and the DSES scale were examined to assess convergent validity. Correlations were high (Factor 1, *r*=0.67, *P*<.001 and Factor 2, *r*=0.75, *P*<.001), indicating the scales were tapping into similar but different concepts.

As predicted, those who did use technology to support self-management scored more highly on both new DMST-Q scales. For Factor 1, those using technology to support self-management (n=92) had a mean score of 78.81 (SD 16.64), while those who did not use technology (n=158) scored 72.60 (SD 18.42, t_248_=3.09, *P*=.002). For Factor 2, those using technology to support self-management (n=92) had a mean score of 80.21 (SD 14.61), while those who did not use technology (n=158) scored 72.52 (SD 18.73, t_248_=3.38, *P*<.001).

Factor 1 and 2 also demonstrated good test-retest reliability with ICC values, for those who had indicated their health had remained the same compared to two weeks ago (n=113), equal to 0.78 (*P*<.001) and 0.74 (*P*<.001), respectively.

## Discussion

### Principal Findings

This paper reports the development of a new instrument, the DSMT-Q, to assess self-management in people living with type 2 diabetes using web-based and mHealth technologies to manage their health. Phase 1 used patient and expert feedback to reduce and refine 36 candidate items to 21 items. Phase 2 further refined items using EFA and confirmed the presence of two sub-scales. The first sub-scale contained seven items and was entitled “Understanding individual health and making informed decisions.” Understanding individual health to make informed decisions was found to be extremely important in the preliminary qualitative work to support this research [19]. It is further supported by other diabetes research that links logging, visualizing, and understanding individual health to acquire new knowledge and make changes to behavior [38]. Similar conclusions can also be found in other condition groups, including COPD, where mHealth applications have also been used to support self-management [39].

The second subscale contained six items and was entitled “Confidence to reach and sustain goals.” Although the preliminary qualitative work carried out to inform this instrument supports the grouping of these items, it is also supported through early research, which links the potential of web-based interventions to patient empowerment [40]. Gaining confidence and taking ownership to reach personal goals through monitoring physical activity on a mobile app has also been demonstrated among patients in the primary care setting [41].

Statistical analyses confirmed the DSMT-Q subscales were highly related to the DSES scale scores and therefore providing evidence of similar, yet distinct constructs. As expected, no significant differences were found for sex or age. Respondents who indicated that they did use technology to manage their health scored more highly on both scales indicating that the items can differentiate between technology and nontechnology users. Internal and external reliability was demonstrated for both scales.

The methods used in this study enabled input from both people living with type 2 diabetes and experts during the refinement of the new instrument. Incorporating the patient throughout the stages of instrument development is essential to ensure the user’s perspective is accurately reflected [18,42], and the inclusion of experts helped to ensure that the instrument would be of use in a range of applied settings.

Questionnaire items were also designed to be used in a comparator, nonintervention group to maximize utility of the measure. Although the items are based on themes identified as relevant to the use of web-based and mHealth technologies, they are also applicable and worded appropriately for those who are not using technology to manage their health. As such, this instrument may be used in a variety of contexts where a comparator group receives standard care or other technology or non–technology-based resources. In contrast with other technology-specific instruments that include references to a specific device or website [43], the wording of the items allows for the responder to use multiple resources, for example, mobile apps plus wearable devices.

### Limitations

There are a few limitations to this study regarding the participant sample. First, Black, Asian, and minority ethnic groups were underrepresented within the sample. Second, it should be noted that the use of survey panels to recruit participants for surveys is in its relative infancy in the patient-reported outcome setting; however, it is a method that has been incorporated into other research settings, such as health economics [44]. With regard to measurement properties, further longitudinal research is required to demonstrate the instrument’s sensitivity to change.

### Conclusions

This paper reports two phases of the development of a new instrument—the DSMT-Q. Analyses confirmed good psychometric properties in the DSMT-Q scales. This tool is fully compliant with relevant regulatory bodies, such as the FDA and EMA, and will facilitate the measurement of self-management in people living with type 2 diabetes using web-based or mHealth technologies.

## Abbreviations

CBG: continuous blood glucose
DSES: Diabetes Self-Efficacy Scale
DSMT-Q: Diabetes Self-Management and Technology Questionnaire
EFA: exploratory factor analysis
HbA_1c_: glycated hemoglobin
ICC: intraclass correlation coefficient
KMO: Kaiser–Meyer–Olkin
NHS: National Health Service
